# Antagonistic Action of *Bacillus subtilis* Strain SG6 on *Fusarium graminearum*

**DOI:** 10.1371/journal.pone.0092486

**Published:** 2014-03-20

**Authors:** Yueju Zhao, Jonathan Nimal Selvaraj, Fuguo Xing, Lu Zhou, Yan Wang, Huimin Song, Xinxin Tan, Lichao Sun, Lancine Sangare, Yawa Minnie Elodie Folly, Yang Liu

**Affiliations:** 1 Institute of Agro-products Processing Science and Technology, Chinese Academy of Agricultural Sciences, Beijing, P. R. China; 2 Key Laboratory of Agro-products Processing, Ministry of Agriculture, Beijing, P. R. China; Loyola University Medical Center, United States of America

## Abstract

*Fusarium graminearum* causes *Fusarium* head blight (FHB), a devastating disease that leads to extensive yield and quality loss of wheat and barley. Bacteria isolated from wheat kernels and plant anthers were screened for antagonistic activity against *F. graminearum*. Based on its *in vitro* effectiveness, strain SG6 was selected for characterization and identified as *Bacillus subtilis*. *B. subtilis* SG6 exhibited a high antifungal effect on the mycelium growth, sporulation and DON production of *F. graminearum* with the inhibition rate of 87.9%, 95.6% and 100%, respectively. In order to gain insight into biological control effect *in situ*, we applied *B. subtilis* SG6 at anthesis through the soft dough stage of kernel development in field test. It was revealed that *B. subtilis* SG6 significantly reduced disease incidence (DI), FHB index and DON (P≤0.05). Further, ultrastructural examination shows that *B. subtilis* SG6 strain induced stripping of *F. graminearum* hyphal surface by destroying the cellular structure. When hypha cell wall was damaged, the organelles and cytoplasm inside cell would exude, leading to cell death. The antifungal activity of SG6 could be associated with the coproduction of chitinase, fengycins and surfactins.

## Introduction

*Fusarium graminearum* causes Fusarium head blight (FHB), a widespread destructive disease of small grained cereals, resulting in yield loss [1]–[3]. Also FHB causes the reduction of grain quality, by producing a range of toxic metabolites, especially deoxynivalenol (DON) which poses a serious threat to animal health and food safety [4], [5].

Though some success in controlling FHB can be expected by plowing fields to remove or bury crop residues infected with *F. graminearum* after harvest, minimal tillage practices render this method unacceptable [6]. The use of host resistance is an economically and environmentally effective strategy for controlling FHB. Till date, only a few highly resistant wheat cultivars have been identified from different geographic regions, including Asia, South and North America, and Europe [7]–[10]. Foliar fungicides applied at anthesis can be useful in reducing scab [8]. Due to the growing cost of chemical pesticides and increasing awareness about their negative effect, the farmers are looking for alternative substitutes for these products to fulfill the consumers demand on pesticide-free food while maintaining environmental safety.

Biological control of *F. graminearum* has shown promise in previous studies due to their low enviromental impact, and their ability to help reduce growers’ dependency on chemicals, thereby slowing the development of fungicide resistance in pathogen populations [11], [12]. Several bacteria or fungal strains have been reported to have antagonistic effects against *F. graminearum* [13]. Among them, *Bacillus* strains are well-known antibiotic producers, which have advantage over other biocontrol microorganisms due to their inherent property to form endospores and resistance to extreme conditions. The antagonistic effects of *Bacillus* strains have been shown by *in vitro* antibiosis [14] and *in situ* disruption of spikelet infection leading to reduced disease severities [15]–[20], and identifying the lipopeptides [11], [21]. Regarding antimicrobial mechanism study, production of antifungal compounds is thought to be the main mode of action by the antagonistic bacteria.

In an attempt to develop biological control of FHB and DON contamination using antagonistic microorganism, we isolated a *B. subtilis* strain SG6 displaying a strong inhibitory effect on *F. graminearum.* The objective of the present study was to (1) evaluate inhibitory effect of *B. subtilis* strain SG6 on *F. graminearum* mycelial growth, sporulation and DON production; (2) determine the antagonistic efficacy of *B. subtilis* strain SG6 in controlling FHB in field condition; (3) examine the ultrastructural alterations occurring in hypha cells of *F. graminearum* during interaction with *B. subtilis* SG6 by transmission electron microscopy (TEM) and scanning electron microscope (SEM); (4) analyze antifungal peptides to investigate the putative biocontrol mechanism.

## Results

### Isolation and Screening of Bacteria

Totally 136 isolates were obtained from wheat kernels and plant anthers. Of these, 24 isolates showed a wide range of apparent antagonistic activity against *F. graminearum*. Notably, isolate SG6 showed the highest apparent antagonistic activity (Table 1), and was selected for further characterization and investigation.

**Table 1:** Apparent antagonistic activity of isolates against *F. graminearum* D187 on potato dextrose agar.

Strain No.	Isolate No.	Origin	inhibition distance^a^
1	SD1	wheat kernels collected from Shandong Province	++
2	SD2	wheat kernels collected from Shandong Province	+
3	SD3	wheat kernels collected from Shandong Province	++
4	SD4	wheat kernels collected from Shandong Province	++
8	SD8	wheat kernels collected from Shandong Province	++
9	ZZ2	wheat kernels collected from Hebei Province	++
10	ZZ3	wheat kernels collected from Hebei Province	++
11	ZZ4	wheat kernels collected from Hebei Province	++
12	HB1	wheat kernels collected from Hubei Province	++
14	CR1	Anthers of Chinese Rose	++
15	SG3	Anthers of luffa	+
16	SG5	Anthers of luffa	+
17	**SG6**	Anthers of luffa	+++
18	ZJ1	Anthers of henna	++
19	BE1	Anthers of beans	++
20	WG1	Anthers of pumpkin	++
21	BJ1	wheat kernels collected from Beijing	++
22	BJ2	wheat kernels collected from Beijing	++
23	BJ3	wheat kernels collected from Beijing	++
24	BJ4	wheat kernels collected from Beijing	++

a Antagonistic activity was assayed in dual-culture method, then averaged, and assigned to one of three categories: +, slight inhibition with a discernible (<1 mm) clear zone from mycelial growth; ++, moderate inhibition with a 1- to 3-mm clear zone from mycelial growth; and +++, high inhibition with a clear zone of >3 mm from mycelial growth.

### Characterization and Identification of Isolate SG6

The morphological, biochemical and physiological characteristics of strain SG6 were determined. The cells are Gram-positive, endospore-forming, aerobic, rods. Oxidase reactions, catalase reactions, Voges-Proskauer test, methyl red reaction and nitrite reduction are positive. It was capable of utilizing citrate and hydrolyzed starch and casein. It was able to grow at 50°C or at pH 5.7. According to 16S rRNA gene sequence analysis, it was found that the closest relatives of strain SG6 were *B. subtilis* subsp. *subtilis* NCIB 3610 (99.72%) and *B. siamensis* KCTC 13613 (99.72%). Based on gyrB gene sequence analysis, strain SG6 displayed the highest sequence similarity (99%) to several *Bacillus subtilis* strains, such as strain PY79, 6051-HGW and BEST7003. Strain SG6 was finally identified as *B. subtilis* SG6. The partial 16S rRNA gene and gyrB gene sequences of strain SG6 were submitted to the database of DNA Data Bank of Japan, and the accession numbers are AB858386 and AB909427, respectively.

### In vitro Studies on the Effect of *B. subtilis* SG6 Strain against *F. graminearum*

**Figure 1:**
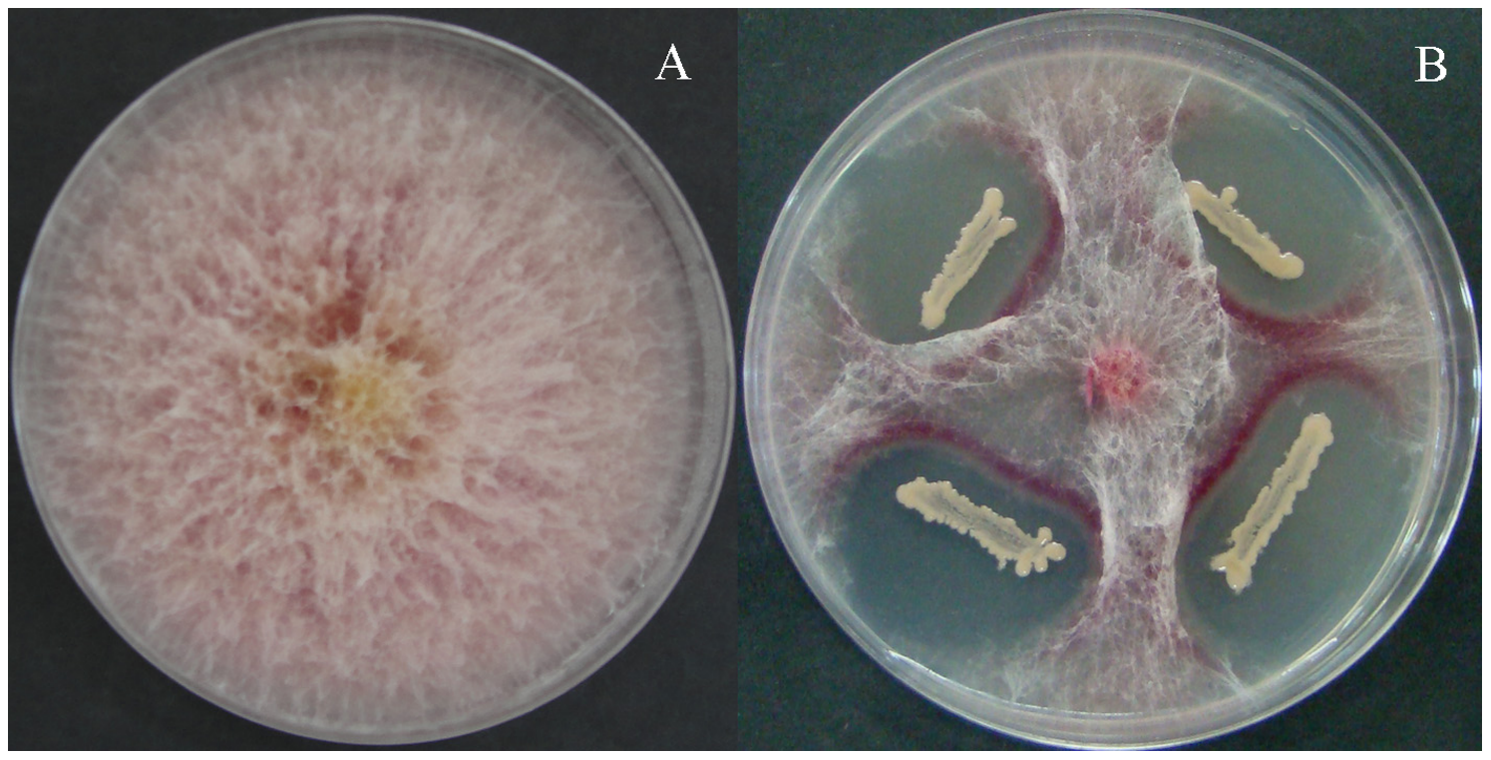
*In vitro* interaction between *B. subtilis* SG6 and *F. graminearum* in dual culture on PDA plate at 5^th^ day after incubation at 28°C (A) A 5-mm agar plug of *F. graminearum* on center of PDA plate and (B) *B. subtilis* SG6 is inoculated on 4 sites of PDA plate with equal distance each other 2.5 cm apart from the colony of *F. graminearum*.

*B. subtilis* SG6 showed a high level of antifungal activity. Hyphal growth of *F. graminearum* was inhibited (Fig. 1). Then the mycelial growth was analyzed with different concentration of *B. subtilis* SG6 (Table 2). It showed that the mycelium diameter of *F. graminearum* was significantly decreased with increase in concentration of *B. subtilis* SG6 in PDA plate, resulting to a gradual increase in inhibition ratio of *F. graminearum*. The inhibition ratio of *F. graminearum* could reach the highest as 87.9% at 10^8^ CFU ml^−1^ concentrations of *B. subtilis* SG6.

**Table 2:** The inhibitory effect of *B. subtilis* SG6 on growth of *F. graminearum* mycelium.

*B. subtilis* concentration (CFU mL^−1^)	CK	10^4^	10^5^	10^6^	10^7^	10^8^
Inhibition ratio percent^a^	0a	72.7b	79.2c	81.3cd	83.7de	87.9e

a Colony radius was measured after 5 days of incubation at 28°C Values followed by the same letter are not significantly different at P≤0.05 according to Fisher’s protected least significant difference (LSD) test.

Further, inhibition of sporulation in *F. graminearum* by *B. subtilis* SG6 strain was significant (Table 3). Stain SG6 at a concentration of 10^4^ CFU ml^−1^ could reduce the spore number of *F. graminearum* by 83.7% compared with the untreated control. With the increase in concentration of *B. subtilis* SG6, the inhibition ratio of sporulation gradually increased. No obvious differences in inhibitory effects between different concentrations were found.

**Table 3:** The inhibitory effect of *B. subtilis* SG6 on sporulation of *F. graminearum*.

*B. subtilis* concentration (CFU mL^−1^)	CK	10^4^	10^5^	10^6^	10^7^	10^8^
Numbers of spore of *F. graminearum* (×10^3^)^a^	99	16	11	5	4	4
Inhibition ratio (%)^b^	0a	83.7b	88.5b	94.7b	95.8b	95.6b

a Numbers of spore were determined after 21 days of incubation at 28°C.

b Values followed by the same letter are not significantly different at P≤0.05 according to Fisher’s protected least significant difference (LSD) test.

### Effects of *B. subtilis* SG6 Strain on Deoxynivalenol Production of *F. graminearum*

When co-cultured with *B. subtilis* SG6, growth of *F. graminearum* D187 was greatly inhibited and ergosterol extracted decreased by 87.18% when compared with the control (Table 4). Meanwhile no DON could be detected, while DON content of *F. graminearum* D187 in the control group was 2.97 μg/mg ergosterol (Table 4). It shows that SG6 could significantly reduce DON production in wheat.

**Table 4:** The inhibitory effect of *B. subtilis* SG6 on DON production of *F. graminearum*.

	DON(μg/g)	ergosterol(μg/g)	DON (μg/mg ergosterol )
CK^a^	0.85±0.24	351.36±44.99	2.97±1.07
S	0	45.16±26.37	0

a CK denoted healthy wheat kernels inoculated with 1 ml spore suspension (10^5^ spores/ml) of *F. graminearum* D187. S denoted healthy wheat kernels inoculated with 1 ml spore suspension (10^5^ spores/ml) of *F. graminearum* D187 and 1 ml *B. subtilis* strain SG6 suspension (10^8^ CFU/ml).

### Effects of *B. subtilis* SG6 Strain on *F. graminearum* in the Field

*B. subtilis* SG6 significantly reduced DI, FHB index and DON in the field trials. Strain SG6 reduced DI by 72.6%, and FHB index by 77.5% compared with the untreated controls. These effects were more pronounced than those of carbendazim, which reduce DI by 8.8% and FHB by 40.2%. Based on the data under field conditions, strain SG6 was more effective than the chemical fungicide Carbendazim widely used in China in reducing DI and FHB index (Table 5). While yield is a widely used parameter for evaluating the efficacy of control of FHB [19], strain SG6 did not show a significant decrease in 100-kernel weight (P>0.05) when compared to the untreated controls. Strain SG6 could reduce DON by 69.1% when compared with the untreated controls. Similar DON reduction effects were found for carbendazim, which reduce DON by 73.2%.

**Table 5:** Influence of *B. subtilis* SG6 on *Fusarium* head blight incited by *F. graminearum* D187 on winter wheat cultivar Shixin 838.

Treatment^d^	DS (%)^a^	DI (%)^b^	FHB index (%)	100-kw (g)^c^	DON(μg/g)
SG6	27.9a	17.2a	4.7a	2.24a	5.41a
Carbendazim	21.8a	57.3b	12.5b	3.77b	4.69a
Sterile distilled water	33.2a	62.8b	20.9c	3.18ab	17.50b

a DS = disease severity.

b DI = disease incidence.

c 100-kw = 100-kernel weight.

d Within columns, means followed by the same lower-case letter are not significantly different (Fisher’s protected least significant difference, P≤0.05).

### Effects of *B. subtilis* SG6 Strain on Ultrastructure of *F. graminearum*

**Figure 2:**
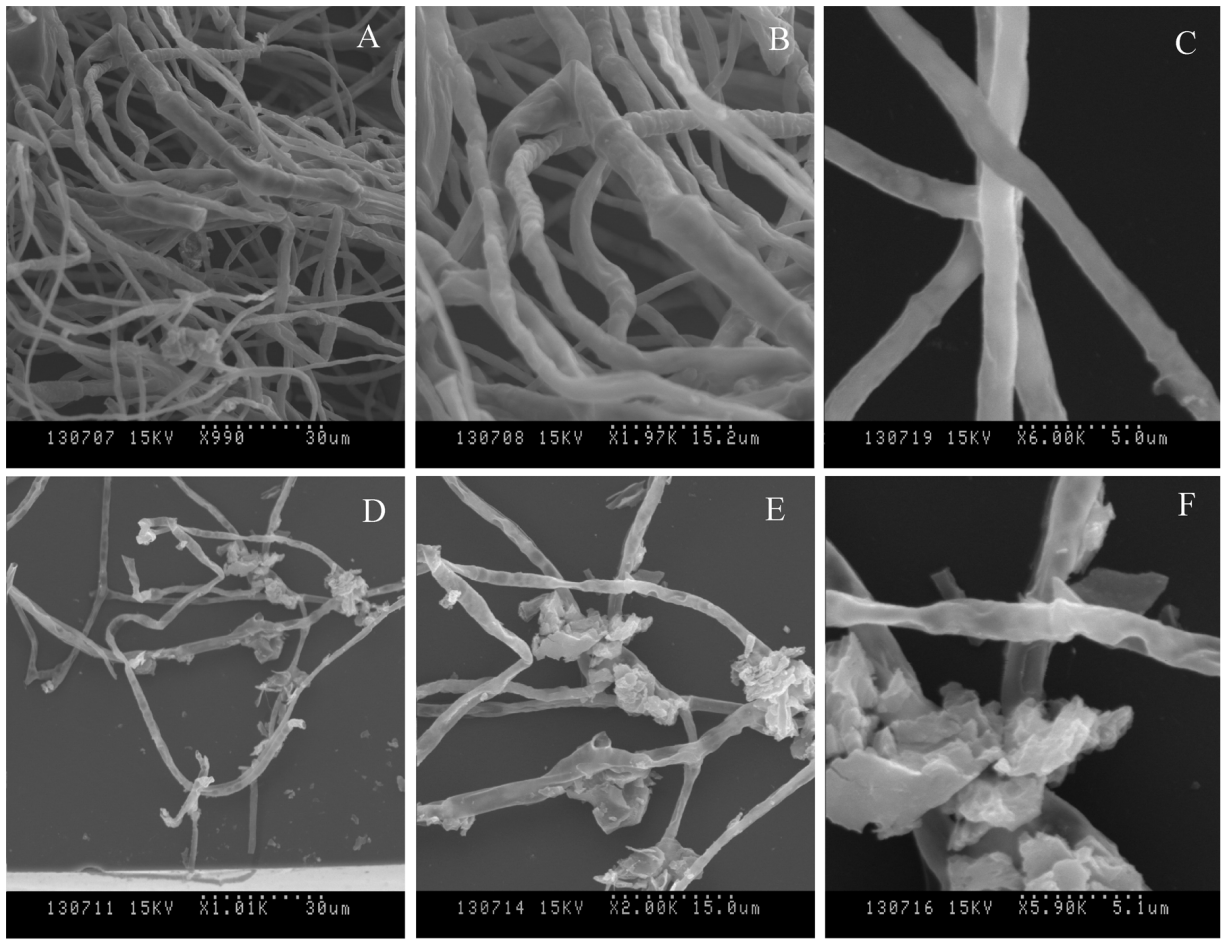
SEM analysis of antagonistic bacteria interacting with hyphae of pathogens on PDA medium at 5^th^ day after incubation at 28°C. A, B, C denoted normal hyphae of *F. graminearum*, D, E, F denoted abnormal hyphae of *F. graminearum.*

Inhibition of *F. graminearum* growth *in vitro* was further complemented by SEM investigations (Fig. 2). Healthy looking hyphae of *F. graminearum* cultured without *B. subtilis* SG6 strain were regular in shape and their surfaces were smooth (Fig. 2A, B and C). Noticeable morphological changes were found in the hyphae of *F. graminearum* in the presence of the antagonistic bacteria. One of the most striking features was a marked hyphal surface flaking (Fig. 2D, E and F). Strain SG6 induced stripping of hyphae surface, leading to debris accumulation or dispersion.

**Figure 3:**
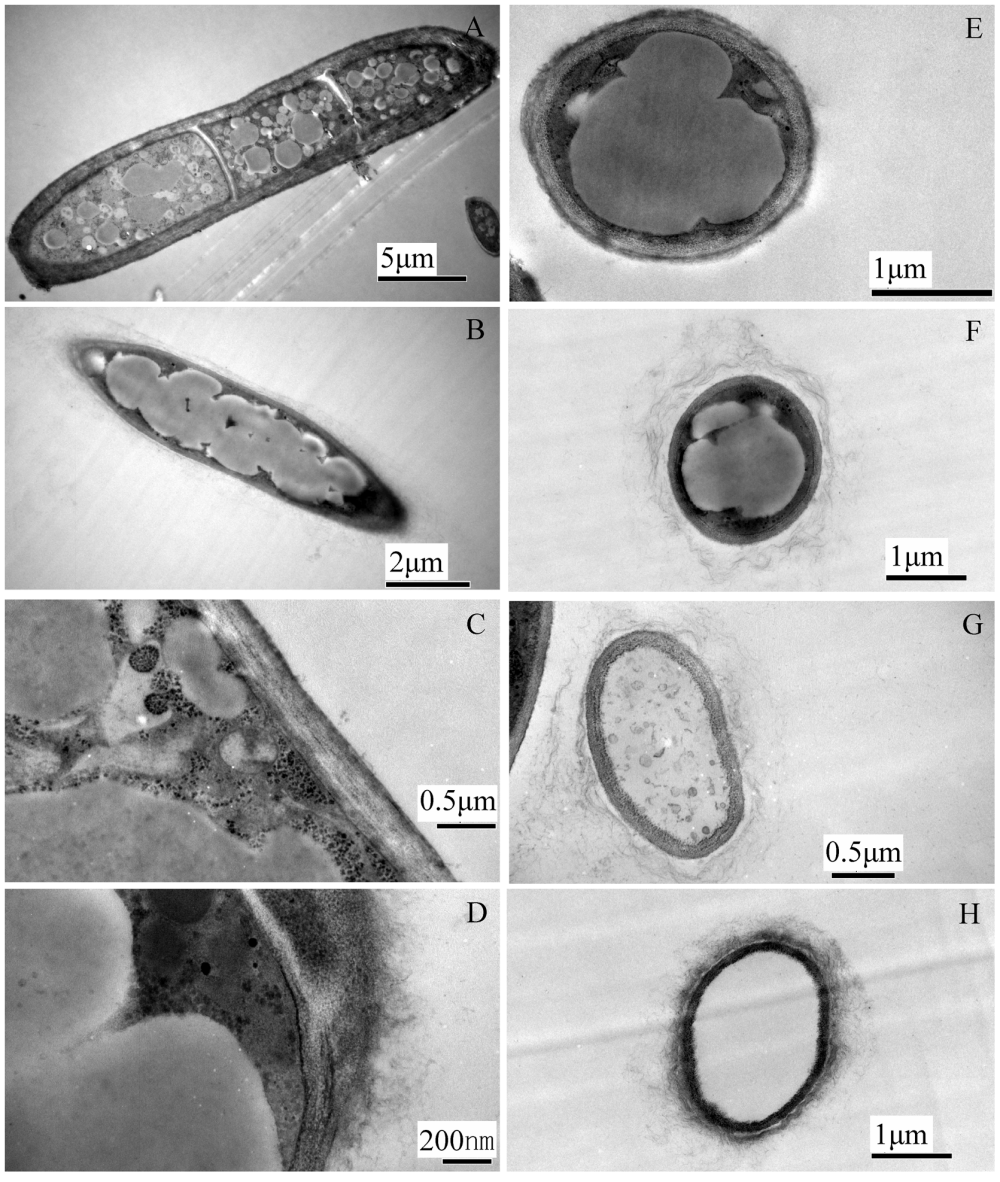
TEM analysis (A–H) of *B. subtilis* SG6 hyphae growing toward colonies of *F. graminearum* at 5^th^ day of interaction on PDA medium. (A) A longitudinal section of control hypha. (B) A longitudinal section of affected hypha. (C) An intact cell wall of a control hypha. (D) A degrading cell wall of affected hypha. (E) A cross-section of a control hypha. (F, G, H) A cross-section of affected hypha.

TEM analysis further elucidated morphological changes of hyphal ultrastructure of *F. graminearum* induced by *B. subtilis* SG6. TEM observation showed that the structure of *F. graminearum* cell remained intact, the enclosing cell wall was well defined and all cell components arranged in order in the untreated controls. However, most treated *F. graminearum* had more or less degradation in cell walls (Fig. 3B, D, F, G and H). The organelles and cytoplasms in the hyphae cell were irregular and degenerated even appeared empty holes (Fig. 3F, G and H). These results indicated that *B. subtilis* SG6 initially break down the cell walls of *F. graminearum*, leading to release of cell contents. Further, chitinase activity of SG6 was detected on chitin-amended media, and clearance halos around and beneath the growth were observed.

### *B. subtilis* SG6 Strain AMP Genes and AMP Profile Analysis

**Figure 4:**
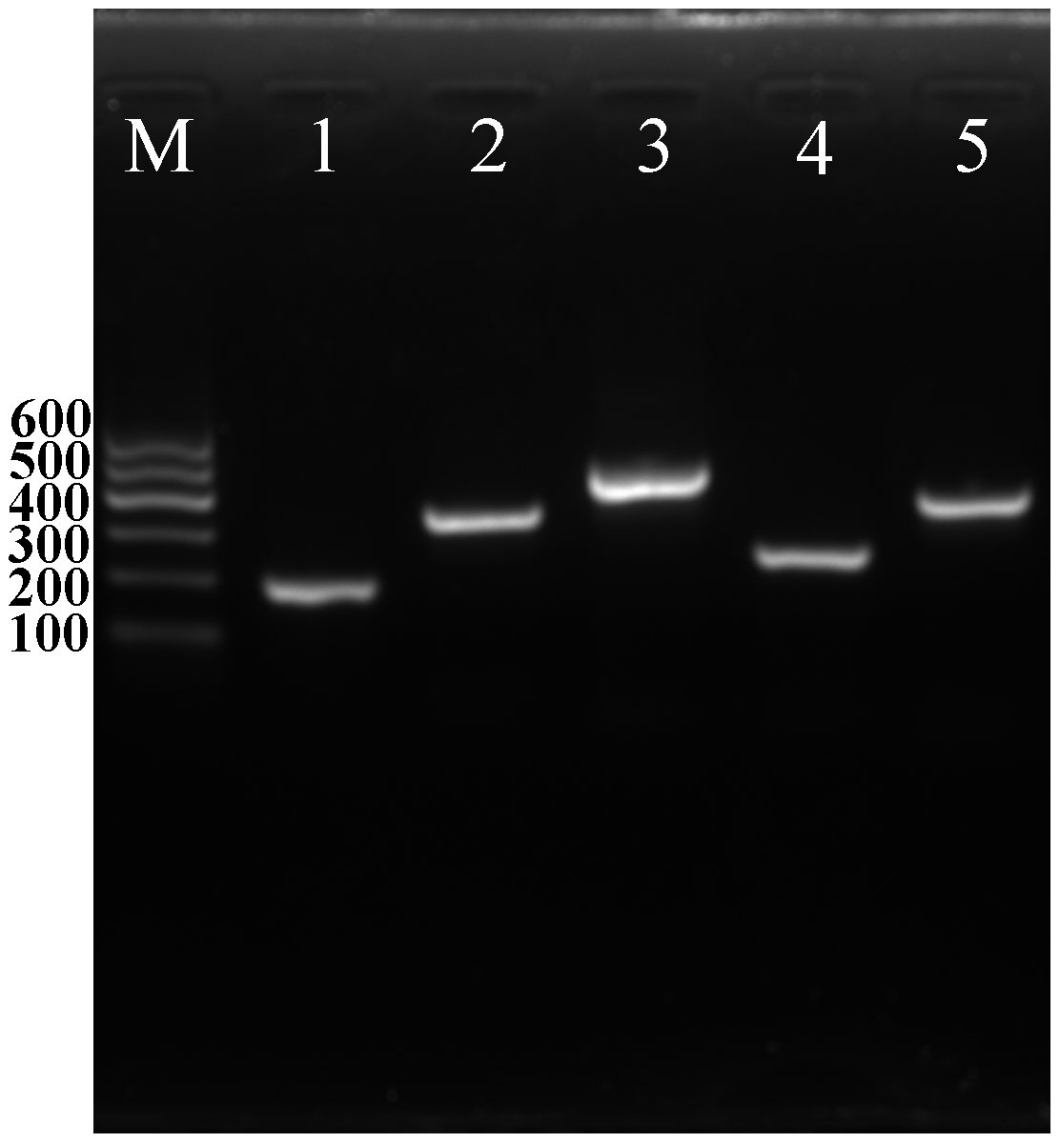
Amplification products of AMP genes. Lane M is a 100-bp ladder. Lane 1 is *srfAA*. Lane 2 is *bmyB*, Lane 3 is *bacA*. Lane 4 is *fenD*. Lane 5 is *ituC*.

AMP biosynthetic genes were reported to be related to biocontrol of plant pathogen in several *Bacillus* [22]–[24]. The presence of five AMP genes markers were checked by PCR (Fig. 4). Amplification of these gene markers showed that each gene had one specific band with the right size.

**Figure 5:**
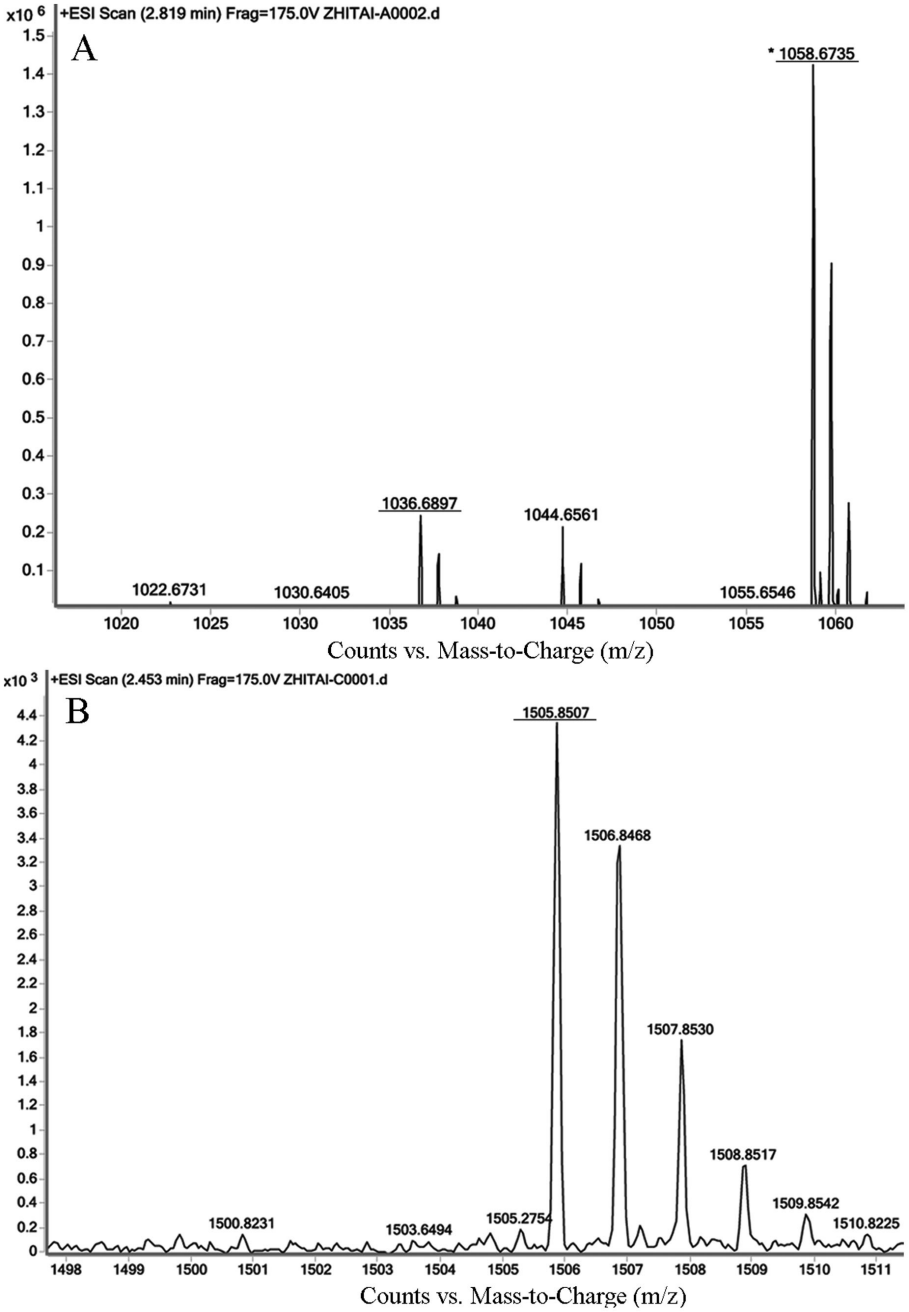
ESI Mass spectra of lipopeptides produced by *B. subtilis* SG6. (A) presents C15 surfactin A, (B) presents C17 fengycin B.

To further characterize the AMP profiles of strain SG6, lipopeptides mixture was precipitated with 6 N HCl and extracted by methanol, the assignment of lipopeptides was on the basis of molecular weight using ESI-MS/CID. A summary of the accumulated lipopeptides is reported in Table 6. The results show stain SG6 mainly produce surfactins and fengycins. The mass spectra of several typical lipopeptides are shown in Figure 5. The masses of the [M+Na]^+^ molecular ions at m/z 1030.6, 1058.7, 1072.7 and 1086.7 differed by 14 Da, suggesting that they are homologous molecules.

**Table 6:** Assignments of major m/z peaks observed in mass spectra of lipopeptides from *B. subtilis* SG6.

Type	m/z	assignments	reference
surfactin	1008.7	surfactin A C13 [M+H]^+^	[64]
		surfactin B C14 [M+H]^+^	
	1022.7	surfactin A C14 [M+H]^+^	
		surfactin B C15 [M+H]^+^	
	1030.6	surfactin A C13 [M+Na]^+^	
		surfactin B C14 [M+Na]^+^	
	1036.7	surfactin A C15[M+H]^+^	
	1044.7	surfactin A C14 [M+Na]^+^	
		surfactin B C15 [M+Na]^+^	
	1050.7	surfactin A C16[M+H]^+^	
	1058.7	surfactin A C15[M+Na]^+^	
	1072.7	surfactin A C16[M+Na]^+^	
	1086.7	surfactin A C17[M+Na]^+^	This study
fengycin	1463.8	C16 fengycin A [M+H]^+^	[62], [64]
	1477.8	C17 fengycin A [M+H]^+^	
	1491.8	C18 fengycin A [M+H]^+^	
	1505.8	C17 fengycin B [M+H]^+^	

## Discussion

In the recent years, various *Bacillus* sp. stains like *B. subtilis*, *B. atrophaeus*, *B. amyloliquefaciens*, *B. cereus*, *B. licheniformis* and *B. pumilis* were used as potential biocontrol agents against different *Fusarium* sp. [25], [26], [27]. Among those, several *B. subtilis* strains had the potential for biocontrol against *F. graminearum* [15]–[20]. Understanding the mode of action between *B. subtilis* and *F. graminearum* is important for developing *B. subtilis* as a successful biological control agent.

It seems that inhibition of hyphal growth is the main pattern of inhibition of *Bacillus* stains against *Fusarium* pathogens. For example, *B. subtilis* EU07 strain could inhibit *F. oxysporum* f. sp. *radicis-lycopersici* growth by 64% [28]. *B. subtilis* strains reduced mycelial growth of *F. solani* by 34.4% [29]. Studies by Chan et al. [14] showed that *B. subtilis* D1/2 showed the inhibition against *F. graminearum, F. subglutinans,* and *F. verticilliodes* with the wider target spectrum. Besides, inhibition of pathogen fungi sporulation could also be observed in some *Bacillus* strains. Dihazi et al. [30] showed in his study on the 5^th^ day *B. amyloliquefaciens* inhibited the sporulation of *F. oxysporum* to 86%. In our study, *B. subtilis* SG6 could effectively inhibit both growth and sporulation of *F. graminearum*.

Selection of antagonists that not only inhibit of pathogen growth and sporulation but also reduce DON production is critical to biocontrol of FHB [31]. More and more studies have considered the importance of reduction in DON production by antagonistic stains. A concurrent selection method for microbial suppression of *F. graminearum*, *Fusarium* head blight and deoxynivalenol in wheat was established by He et al. [32]. 9 isolates screened in Argentina were able to reduce the growth of *F. graminearum* and the production of DON on irradiated wheat grains by 60–100%, and in greenhouse conditions they could significantly reduce DON content in spikes by 32–100% compared to the control treatment [31]. Similar results have been observed in our study. SG6 could significantly reduce DON production in wheat DON assay in lab, but also reduce DON by 69.1% when compared with the untreated control in field test.

*B. subtilis* strains produce a broad spectrum of antimicrobial compounds, including predominantly peptides as well as a couple of non-peptidic compounds such as polyketides, an aminosugar, and a phospholipid [33]. The antifungal effects might have been due to one or more antifungal compounds produced by this biocontrol agent. Chitin is a common constituent of fungal cell walls [34]. SG6 could induce cell wall degradation of *F. graminearum* D187 based on the ultrastructural analysis (Fig. 3D). SG6 could produce chitinase on chitin-amended media. It indicates that SG6 could break down cell wall of *F. graminearum* D187 by producing chitinase. The cell wall of fungi provides both protective and aggressive functions. If removed or weakened, the fungi die unless they are osmotically protected [35]. Secretion of chitinase could be involved in biocontrol of *F. graminearum* in SG6.

Antimicrobial peptides produced by *Bacillus* spp. have been implicated in the biocontrol of several plant pathogens [33], [36], [37]. The presence of AMP biosynthetic genes has been linked to biocontrol of plant pathogens in several *Bacillus* strains [38]–[40]. Presence of five AMP genes (*bmyB*, *fenD*, *ituC*, *srfAA* and *bacA* ) in strain SG6 was checked by PCR. The result of electrophoresis showed that all the five genes exist in SG6. It indicates the presence of the five genes in strain SG6 could be due to the benefit provided by complementary mechanisms of action among the gene products [41].

Lipopeptides profile of strain SG6 had been analyzed. Fengycins and surfactins are the prominent products of strain SG6 when it is cultured in NB for 72 h. Fengycins are cyclic lipodecapeptides which specifically inhibits against filamentous fungi [42]. Fengycins have also been identified as the prominent lipopeptides in other *B. subtilis* strains acting against *F. graminearum* [43], [44]. Possibly fengycins affect the cell membrane of *F. graminearum* to alter its permeability, resulting in release of cell contents (Fig. 3G, H). Surfactins could synergistically impact the anti-fungal activity of other lipopeptides [45]. The simultaneous production of fengycins and surfactins would be important for the efficiency of *F. graminearum* control by strain SG6.

As known, the effectiveness of biological control in the field tests depends on the antagonist dose, the carbon to nitrogen ratio of the antagonist production medium and the wheat cultivar utilized [19]. Further systematic field study is necessary to study under different conditions to compare and evaluate the efficacy of strain SG6 at a larger level.

## Materials and Methods

### Ethics Statement

Specific permission was not needed for our field studies. The strains used in our field study were isolated from natural environment. *B. subtilis* strain SG6 was isolated for anthers of luffa grown in yard in Beijing, while *F. graminearum* D187 was isolated from *Fusarium*-infected wheat kernel collected from Hebei Province (the place of our further field test). No transgenic or created mutant microbes have been used in our study. Also we confirm that the field studies did not involve endangered or protected species.

### Strains, Culture Media and Conditions

*F. graminearum* D187, from the culture collection of our lab, was primarily grown on PDA at 28°C under white fluorescent light. Nutrient broth (NB; Disco) was used at 3 ml per 17×100 mm tube for preparing bacterial cultures on an incubator shaker at 28°C and 200 rpm. Agar (Aobox, Beijing, China) at 15 g/L was added to solidify the liquid media. Bacteria strains were recovered from storage in 10% glycerol at −80°C by briefly warming the vial at room temperature and streaked onto Nutrient Agar (NA) plate. To obtain a large amount of bacterial culture supernatant for cyclic lipopeptides, the bacterium was grown in 2 L conical flasks each containing 0.5 L of NB. The culture was started with a 1% inoculum and incubated at 28°C and 200 rpm for 48 h. After the cells were separated by centrifugation, the supernatant was filter sterilized by using a 500-mL Stericup™ fitted with a 0.22 μM GP Express membrane (Millipore Corp., Bedford, Mass.).

### Antifungal Bacteria Isolation

Altogether 10 wheat kernel samples were collected from Shandong, Hebei, Beijing, Hubei Provinces in 2011(Table S1). Anthers of luffa, bean, Chinese Rose and pumpkin were collected in summer of 2011 in Beijing (Table S1). Bacteria from wheat kernels and plant anthers were isolated according to Hartnett et al. and Khan et al. [15], [46]. Thereafter, 1 ml of cell suspension was serially diluted. Dilutions were plated onto NA plates.

### Morphological and Physiological Properties of Strain SG6

General physiological and biochemical tests were carried out using previously described methods, including Gram-type, morphology, growth properties, catalase and oxidase activities, methyl red reaction, Voges-Proskauer reaction, nitrate reduction and anaerobic growth [47].

### Phylogenetic Identification of Strain SG6 based on 16S rRNA Gene and gyrB Gene Sequence Analysis

Genomic DNA of strain SG6 was extracted using the method described previously [48]. Universal primer sets (27F and 1492R; UP1 and UP2r) were used to amplify the 16S rRNA gene and gyrB gene [49], [50]. The nucleotide sequences were determined by direct sequencing and compared with available 16S rRNA gene sequences in EZTAXON (http://eztaxon-e.ezbiocloud.net/) and gyrB gene in the GenBank database using the BLAST program [51]. Strain SG6 (CGMCC No. 7621) was registered by the China Committee for Culture Collection of Microorganisms.

### Antagonism Assay

Antagonistic effect of isolates was evaluated by a dual-culture assay using relative growth of *F. graminearum* [14], [52]. A 5-mm agar plug cut from an agar culture of D187 was seeded at the center of the PDA plate, and isolates were inoculated at 4 equidistance sites 2.5 cm from the centre. Other plates were inoculated with same size plug of *F*. *graminearum* colony in the absence of test stain as the control. All treatments were replicated triplicates and the plates were incubated at 28°C for 5 days. And then the antagonistic effect of test strains on *F*. *graminearum* D187 was observed.

### Preparations of Bacteria and Pathogen Spore Suspensions

Cells of *B. subtilis* strain SG6 were obtained by rolling a sterile cotton swab on the 36 to 48 h culture and suspended in isotonic saline water (0.85% NaCl). Then the cell suspension was diluted from 10^9^ to l0^5^ CFU/ml as a stock suspension with isotonic saline water (0.85% NaCl) [21], [52].

*F. graminearum* D187was grown in a 250 ml flask with 100 ml of CMC medium (1.5 g of CMC, 0.05 g of NH_4_NO_3_·7H_2_O, 0.1 g of yeast extract and 100 ml of H_2_O ) on a rotary shaker at 200 rev min^−1^ at 25°C for 3 to 5 days to produce macroconidia [53]. A macroconidia suspension was prepared by filtering the culture through sterile filter paper to remove mycelia. The concentration of the suspension was adjusted to 10^5^ conidia/ml.

### Effect of *B. subtilis* Strain SG6 on Mycelial Growth of *F. graminearum*

PDA (1/9 diluted) medium was combined with *B. subtilis* SG6 cells at concentrations of 10^8^, 10^7^, 10^6^, 10^5^ and 10^4^ CFU ml^−1^ and isotonic saline water (0.85% NaCl) was used as a control in 9∶1 proportions. A 5-mm agar plug from an actively growing mycelium of *F. graminearum* D187 was placed on the center of the test plate [54]. All the plates were incubated for 5 d at 28°C Experiment was performed in triplicate. The diameters of fungal colonies were measured. The inhibition ratios of mycelium growth of *F. graminearum* D187 were calculated with the following formula [52].

Inhibition ratio (%) = (*C* − *E*)*/C*×100%, where *C* is the diameter of the control colony and *E* is the diameter of the treatment colonies.

### Effect of *B. subtilis* Strain SG6 on Sporulation of *F. graminearum*

5-mm agar plugs of *F. graminearum* D187 were placed on the center of SNA plates that contained 5 concentrations of *B. subtilis* SG6 cells (10^8^ CFU ml^−1^, 10^7^ CFU ml^−1^, 10^6^ CFU ml^−1^, l0^5^ CFU ml^−1^ and 10^4^ CFU ml^−1^) and isotonic saline water (0.85% NaCl) as a control. All treated sets were incubated at 28°C for 21 d. At the 22^th^ day, 5 ml of isotonic saline water (0.85% NaCl) containing 0.01% (v/v) Tween 80 was added to the plate and the mature spores were harvested with a pipette. The volume of spore suspension was adjusted to 5 ml with 0.01% Tween 80 and dispersed by vortexes, and the spore concentration was determined by a haemocytometer. The inhibition ratios of sporulation of *F. graminearum* were calculated with the following formula: Inhibition ratio (%) = (C − E)/C×100%, where C is the numbers of sporulation of the control groups and E is the numbers of sporulation of the experimental group [52]. All treatments were replicated 4 times.

### Effect of *B. subtilis* Strain SG6 on DON Production of *F. graminearum*

A 50 g aliquot of healthy wheat kernels was sterilized and inoculated with 1 ml spore suspension (10^5^ spores/ml) of *F. graminearum* D187 and 1ml *B. subtilis* strain SG6 suspension (10^8^ CFU/ml). As control, 1 ml spore suspension (10^5^ spores/ml) of *F. graminearum* D187 and 1 ml NB were inoculated. After incubation at 25°C for 20 days, subsequent DON extraction and analysis was performed as described by Bluhm et al. [55]. Ergosterol levels were used to normalize DON content per fungal mass. All treatments were replicated 3 times.

### Hyphal Cell Preparation for Ultrastructural Study

One hundred microliters of spore suspensions of *F. graminearum* D187 (about 10^5^ conidia ml^−1^) were spread uniformly on the surface of each PDA plate contained *B. subtilis* SG6 cells at the concentration of 10^8 ^CFU ml^−1^ and 0.85% NaCl as a control. Plates were placed at 28°C After 5 d, *F. graminearum* D187 hyphae were harvested for SEM and TEM.

### Scanning Electron Microscopy (SEM)

Hyphae were fixed in 2% glutaraldehyde for 4 h at room temperature, rinsed 4 times with phosphate buffer (0.1 M) and subsequently fixed with 1% osmium tetraoxide for 2 h at 20°C The hyphae were dehydrated in a graded series of ethanol concentrations (30%, 50%, 70%, 80%, 90% and 100%) for 15 min each, CO_2_ dried (Leica CPB 030) and sputter coated with gold palladium in a Nanotech sputter coating apparatus (HITACHI IB-5, Japan) [52], [56], [57]. Samples were kept in a desiccator until examination with an SEM (HITACHI, S-570, Japan) operated at 15 kV.

### Transmission Electron Microscopy (TEM)

Similar procedure (in section ‘Scanning electron microscopy’) was used until hyphae dehydrated. After dehydration, the samples were embedded in Epon812 and ultrafine sections (80 nm) of tissue were prepared at room temperature using an ultra-microtome LeicaUC6 and a glass knife. Once the tissue had been mounted on a copper grid, poststaining was carried out (uranyl acetate for 30 min and lead citrate for 20 min) [52], [57]. Samples were kept in a desiccator until examination with a TEM (HITACHI, H-7500, Japan) operated at 80 kV.

### Field Disease Management Trials

Field experiment was conducted at Lianjiazhuang Village, Songlindian Town, Zhuozhou, Hebei Province, P. R. China (E39°23′, N115°56′), in 2012. The FHB intermediately resistant wheat “Shixin 838” was used to test the efficacy of *B. subtilis* SG6 and compared with carbendazim. Experiments were arranged as one-factor factorial design with triplicates. Plants were grown in 10-row plots, 2 m long with 10 cm row spacing. Plots were fertilized based on soil test recommendations. Appropriate herbicides for efficient weed control were applied [58].

At anthesis (Zadoks growth stage 65), wheat spikes were sprayed with 300 ml suspension of strain SG6 at a concentration of 10^8^ CFU ml^−1^, 0.9 g/L carbendazim, or sterile distilled water immediately before treating heads with 300 ml suspension containing 2×10^5^ CFU ml^−1^ conidia of *F. graminearum* D187. At each application, the suspension was sprayed evenly on to the spikes in each plot using a polyethylene compressed air sprayer (Yuanhua Sprayer Inc., Taizhou, Zhejiang Province, P.R. China). The treatments were applied in late afternoon approximately 2h before sunset [13], [58]. At the soft dough stage, plot disease severity for a population of approximately 300 spikes per plot was estimated for both incidence (percentage of infected spikes) and severity (percentage of infected spikelets of the diseased spikes) [16]. An FHB index (incidence×severity/100) was derived to give an assessment of plot disease severity [59]. 100-kernel weight was determined after harvest [13], [16].

For each plot, 30 g seed sample was taken and ground to a fine powder and stored in paper bags at room temperature. From each ground sample, a 5 g subsample was used for DON analysis. The concentration of DON was determined according to the method reported by Maragos [60] and Liu [61] with few modifications, and toxin determination was quantified by HPLC/UV.

### Antimicrobial Peptide (AMP) Gene PCR Assays and AMP ESI-MS/CID Spectrometric Analysis

Primers were developed according to sequences chosen from the coding regions of *bmyB* (bacillomycin L synthetase B), *fenD* (fengycin synthetase), *ituC* (iturin A synthetase C), *srfAA* (surfactin synthetase subunit 1) and *bacA* (bacilysin biosynthesis protein) (Table S2) [41].

PCR was carried out in a total volume of 50 μl containing 25 μl 1× Go Tag ® Colorless Master Mix (Promega), 0.4 μM of each primer, and 5 μl of genomic DNA. The cycling conditions for the amplification of all targets were as follows: 95°C for 4 min, 40 cycles of 94°C for 1 min, annealing temperature for 1 min, and 70°C for 1 min. A final extension step at 70°C for 10 min was followed by a 4°C soak. The annealing temperature was set to 58°C for *fenD*, *ituC*, *srfAA* and *bacA*, to 55°C for *bmyB* [41].

AMP was collected and ESI-MS/CID analysis using methods similar to those previously described [21], [62]. Briefly, lipopeptides were precipitated from cell-free supernatants with 6 N HCl, and extracted with dichloromethane. After evaporation, the recovered materials were re-dissolved in methanol. AMP extract was subjected to the analyzed HPLC and ESI-MS/CID spectrometric analysis.

### Production of Cell Wall Degrading Enzyme

The qualitative assay for chitinase production was performed according to the method described by Marten et al. [25]. Strain SG6 was inoculated as single streak on the chitin containing medium, the plates were incubated at 28°C and clearance halos around and beneath the growth indicating the enzymatic degradation was observed and measured after 5–10 days.

### Statistical Analyses

The mycelia radius and the number of spores were subjected to analysis of variance without transformation. Variance was stabilized using the arcsine square root transformation for DS, IS and FHB index and logarithmic transformation of DON values when needed [63]. Means were separated at P≤0.05 using Fisher’s protected least significance difference test (SPSS Statistics, ver. 17.0, IBM).

## Supporting Information

Table S1 **Information for collected samples.**
(DOCX)

Table S2 **Primers used for AMP amplification in this study.**
(DOCX)

## References

[1] O'Donnell K, Kistler HC, Tacke BK, Casper HH (2000) Gene genealogies reveal global phylogeographic structure and reproductive isolation among lineages of *Fusarium graminearum*, the fungus causing wheat scab. Proc Natl Acad Sci U S A 97: 7905–7910. 10869425 10.1073/pnas.130193297 PMC16643

[2] O’Donnell K, Ward TJ, Geiser DM, Corby Kistler H, Aoki T (2004) Genealogical concordance between the mating type locus and seven other nuclear genes supports formal recognition of nine phylogenetically distinct species within the *Fusarium graminearum* clade. Fungal Genet Biol 41: 600–623. 15121083 10.1016/j.fgb.2004.03.003

[3] Starkey DE, Ward TJ, Aoki T, Gale LR, Kistler HC, et al (2007) Global molecular surveillance reveals novel Fusarium head blight species and trichothecene toxin diversity. Fungal Genet Biol 44: 1191–1204. 17451976 10.1016/j.fgb.2007.03.001

[4] Snijders CHA (1990) Fusarium head blight and mycotoxin contamination of wheat, a review. Neth J Plant Path 96: 187–198.

[5] Tuite J, Shaner G, Everson RJ (1990) Wheat scab in soft red winter wheat in Indiana in 1986 and its relation to some quality measurements. Plant Dis 74: 959–962.

[6] Schisler DA, Khan NI, Boehm MJ (2003) Yeasts for reducing fusarium head blight in cereals and selection thereof. Google Patents.

[7] Hao C, Wang Y, Hou J, Feuillet C, Balfourier F, et al (2012) Association mapping and haplotype analysis of a 3.1-Mb genomic region involved in Fusarium head blight resistance on wheat chromosome 3BS. PLoS ONE 7: e46444. 23071572 10.1371/journal.pone.0046444 PMC3465345

[8] Snijders C (1995) Breeding for resistance to Fusarium in wheat and maize.

[9] Liu S, Anderson J (2003) Marker assisted evaluation of Fusarium head blight resistant wheat germplasm. Crop Sci 43: 760–766.

[10] Yu J, Bai G, Cai S, Dong Y, Ban T (2008) New Fusarium head blight-resistant sources from Asian wheat germplasm. Crop Sci 48: 1090–1097.

[11] Crane J, Gibson D, Vaughan R, Bergstrom G (2013) Iturin Levels on Wheat Spikes Linked to Biological Control of Fusarium Head Blight by *Bacillus amyloliquefaciens* . Phytopathology 103: 146–155. 23075168 10.1094/PHYTO-07-12-0154-R

[12] Jochum C, Osborne L, Yuen G (2006) Fusarium head blight biological control with *Lysobacter enzymogenes* strain C3. Biol Control 39: 336–344.

[13] Xue A, Voldeng H, Savard M, Fedak G, Tian X, et al (2009) Biological control of fusarium head blight of wheat with *Clonostachys rosea* strain ACM941. Can J Plant Pathol 31: 169–179.

[14] Chan Y-K, McCormick WA, Seifert KA (2003) Characterization of an antifungal soil bacterium and its antagonistic activities against *Fusarium* species. Can J Microbiol 49: 253–262. 12897834 10.1139/w03-033

[15] Khan NI, Schisler DA, Boehm MJ, Slininger PJ, Bothast RJ (2001) Selection and Evaluation of Microorganisms for Biocontrol of *Fusarium* Head Blight of Wheat Incited by *Gibberella zeae* . Plant Dis 85: 1253–1258. 10.1094/PDIS.2001.85.12.1253 30831786

[16] Schisler DA, Khan NI, Boehm MJ, Slininger PJ (2002) Greenhouse and Field Evaluation of Biological Control of Fusarium Head Blight on Durum Wheat. Plant Dis 86: 1350–1356. 10.1094/PDIS.2002.86.12.1350 30818440

[17] Stockwell CA, Bergstrom GC, Luz WC. Biological control of fusarium head blight with *Bacillus subtilis* TrigoCor 1448.; 2001 8–10 December 2001; Michigan State University. 91–95.

[18] Bleakley BH, Ruden KR, Murthy NS, Arens A, Halley S (2012) Trial of the Performance of Selected Biological Control Agents for the Suppression of *Fusarium* Head Blight in South Dakota and North Dakota; 2012 December 4–6, 2012; Wyndham Orlando Resort Orlando, Florida. 7.

[19] Khan N, Schisler D, Boehm M, Lipps P, Slininger P (2004) Field testing of antagonists of Fusarium head blight incited by *Gibberella zeae* . Biol Control 29: 245–255.

[20] Schisler DA, Khan NI, Boehm MJ (2002) Biological control of Fusarium head blight of wheat and deoxynivalenol levels in grain via use of microbial antagonists. Mycotoxins and Food Safety: Springer. 53–69. 10.1007/978-1-4615-0629-4_6 11922099

[21] Dunlap CA, Schisler DA, Price NP, Vaughn SF (2011) Cyclic lipopeptide profile of three *Bacillus subtilis* strains; antagonists of *Fusarium* head blight. J Microbiol (Seoul) 49: 603–609. 10.1007/s12275-011-1044-y 21887643

[22] González-Sánchez MÁ, Pérez-Jiménez RM, Pliego C, Ramos C, De Vicente A, et al (2010) Biocontrol bacteria selected by a direct plant protection strategy against avocado white root rot show antagonism as a prevalent trait. J Appl Microbiol 109: 65–78. 19961545 10.1111/j.1365-2672.2009.04628.x

[23] Joshi R, McSpadden Gardener BB (2006) Identification and Characterization of Novel Genetic Markers Associated with Biological Control Activities in *Bacillus subtilis* . Phytopathology 96: 145–154. 18943917 10.1094/PHYTO-96-0145

[24] Romero D, de Vicente A, Rakotoaly RH, Dufour SE, Veening J-W, et al (2007) The Iturin and Fengycin Families of Lipopeptides Are Key Factors in Antagonism of *Bacillus subtilis* Toward *Podosphaera fusca* . Mol Plant-Microbe Interact 20: 430–440. 17427813 10.1094/MPMI-20-4-0430

[25] Marten P, Smalla K, Berg G (2000) Genotypic and phenotypic differentiation of an antifungal biocontrol strain belonging to *Bacillus subtilis* . J Appl Microbiol 89: 463–471. 11021578 10.1046/j.1365-2672.2000.01136.x

[26] Siddiqui S, Siddiqui ZA, Ahmad I (2005) Evaluation of fluorescent *Pseudomonads* and *Bacillus* isolates for the biocontrol of a wilt disease complex of pigeonpea. World J Microb Biot 21: 729–732.

[27] Li D, Nie F, Wei L, Wei B, Chen Z (2007) Screening of high-yielding biocontrol bacterium Bs-916 mutant by ion implantation. Appl Microbiol Biotechnol 75: 1401–1408. 17549470 10.1007/s00253-007-0951-7

[28] Baysal Ö, Çalışkan M, Yeşilova Ö (2008) An inhibitory effect of a new *Bacillus subtilis* strain (EU07) against *Fusarium oxysporum* f. sp. radicis-lycopersici Physiol Mol Plant Pathol 73: 25–32.

[29] Morsy EM, Abdel-Kawi K, Khalil M (2009) Efficiency of *Trichoderma viride* and *Bacillus subtilis* as bio-control agents against *Fusarium solani* on tomato plants. Egypt J Phytopathology 37: 47–57.

[30] Dihazi A, Jaiti F, Wafataktak, Kilani-Feki O, Jaoua S, et al (2012) Use of two bacteria for biological control of bayoud disease caused by *Fusarium oxysporum* in date palm (*Phoenix dactylifera L*) seedlings. Plant Physiol Biochem (Paris) 55: 7–15. 22480991 10.1016/j.plaphy.2012.03.003

[31] Palazzini JM, Ramirez ML, Torres AM, Chulze SN (2007) Potential biocontrol agents for Fusarium head blight and deoxynivalenol production in wheat. Crop Prot 26: 1702–1710.

[32] He J, Boland GJ, Zhou T (2009) Concurrent selection for microbial suppression of Fusarium graminearum, Fusarium head blight and deoxynivalenol in wheat. J Appl Microbiol 106: 1805–1817. 19298518 10.1111/j.1365-2672.2009.04147.x

[33] Stein T (2005) *Bacillus subtilis* antibiotics: structures, syntheses and specific functions. Mol Microbiol 56: 845–857. 15853875 10.1111/j.1365-2958.2005.04587.x

[34] Cohen-Kupiec R, Chet I (1998) The molecular biology of chitin digestion. Curr Opin Biotechnol 9: 270–277. 9650272 10.1016/s0958-1669(98)80058-x

[35] Latgé J-P (2007) The cell wall: a carbohydrate armour for the fungal cell. Mol Microbiol 66: 279–290. 17854405 10.1111/j.1365-2958.2007.05872.x

[36] Mannanov RN, Sattarova RK (2001) Antibiotics Produced by *Bacillus* Bacteria. Chem Nat Comp 37: 117–123.

[37] Montesinos E (2007) Antimicrobial peptides and plant disease control. FEMS Microbiol Lett 270: 1–11. 17371298 10.1111/j.1574-6968.2007.00683.x

[38] Gonzalez-Sanchez MA, Perez-Jimenez RM, Pliego C, Ramos C, de Vicente A, et al (2010) Biocontrol bacteria selected by a direct plant protection strategy against avocado white root rot show antagonism as a prevalent trait. J Appl Microbiol 109: 65–78. 19961545 10.1111/j.1365-2672.2009.04628.x

[39] Joshi R, McSpadden Gardener BB (2006) Identification and Characterization of Novel Genetic Markers Associated with Biological Control Activities in *Bacillus subtilis* . Phytopathology 96: 145–154. 18943917 10.1094/PHYTO-96-0145

[40] Romero D, de Vicente A, Rakotoaly RH, Dufour SE, Veening JW, et al (2007) The iturin and fengycin families of lipopeptides are key factors in antagonism of *Bacillus subtilis* toward *Podosphaera fusca* . Mol Plant Microbe Interact 20: 430–440. 17427813 10.1094/MPMI-20-4-0430

[41] Mora I, Cabrefiga J, Montesinos E (2011) Antimicrobial peptide genes in *Bacillus* strains from plant environments. Int Microbiol 14: 213–223. 22569759 10.2436/20.1501.01.151

[42] Vanittanakom N, Loeffler W, Koch U, Jung G (1986) Fengycin–a novel antifungal lipopeptide antibiotic produced by *Bacillus subtilis* F-29-3. J Antibiot 39: 888–901. 3093430 10.7164/antibiotics.39.888

[43] Ramarathnam R, Bo S, Chen Y, Fernando WGD, Xuewen G, et al (2007) Molecular and biochemical detection of fengycin- and bacillomycin D-producing *Bacillus* spp., antagonistic to fungal pathogens of canola and wheat. Can J Microbiol 53: 901–911. 17898845 10.1139/W07-049

[44] Romanenko LA, Uchino M, Kalinovskaya NI, Mikhailov VV (2008) Isolation, phylogenetic analysis and screening of marine mollusc-associated bacteria for antimicrobial, hemolytic and surface activities. Microbiol Res 163: 633–644. 19216104 10.1016/j.micres.2006.10.001

[45] Hiraoka H, Asaka O, Ano T, Shoda M (1992) Characterization of *Bacillus subtilis* RB14, coproducer of peptide antibiotics iturin A and surfactin. J Gen Appl Microbiol 38: 635–640.

[46] Hartnett DJ, Vaughan A, van Sinderen D (2002) Antimicrobial-Producing Lactic Acid Bacteria Isolated from Raw Barley and Sorghum. J Inst Brew 108: 169–177.

[47] Xue Y, Zhang X, Zhou C, Zhao Y, Cowan DA, et al (2006) *Caldalkalibacillus thermarum* gen. nov., sp. nov., a novel alkalithermophilic bacterium from a hot spring in China. Int J Syst Evol Microbiol 56: 1217–1221. 16738094 10.1099/ijs.0.64105-0

[48] Marmur J (1961) A procedure for the isolation of deoxyribonucleic acid from micro-organisms. J Mol Biol 3: 208–IN201.

[49] Lane DJ (1991) 16S/23S rRNA sequencing. In: Nucleic Acid Techniques in Bacterial Systematics. Chichester: John Wiley and Sons.

[50] Yamamoto S, Harayama S (1995) PCR amplification and direct sequencing of gyrB genes with universal primers and their application to the detection and taxonomic analysis of *Pseudomonas putida* strains. Appl and Environ Microb 61: 1104–1109. 10.1128/aem.61.3.1104-1109.1995 PMC167365 7793912

[51] Kim OS, Cho YJ, Lee K, Yoon SH, Kim M, et al (2012) Introducing EzTaxon-e: a prokaryotic 16S rRNA gene sequence database with phylotypes that represent uncultured species. Int J Syst Evol Microbiol 62: 716–721. 22140171 10.1099/ijs.0.038075-0

[52] Zhou X, Lu Z, Lv F, Zhao H, Wang Y, et al (2011) Antagonistic action of *Bacillus subtilis* strain fmbj on the postharvest pathogen *Rhizopus stolonifer* . J Food Sci 76: M254–259. 22417435 10.1111/j.1750-3841.2011.02160.x

[53] Witt MF, Hart LP, Pestka JJ (1985) Purification of deoxynivalenol (vomitoxin) by water-saturated silica gel chromatography. J Agric Food Chem 33: 745–748.

[54] Leelasuphakul W, Hemmanee P, Chuenchitt S (2008) Growth inhibitory properties of *Bacillus subtilis* strains and their metabolites against the green mold pathogen *Penicillium digitatum* Sacc. of citrus fruit. Postharvest Biol Tec 48: 113–121.

[55] Bluhm B, Zhao X, Flaherty J, Xu J-R, Dunkle L (2007) RAS2 regulates growth and pathogenesis in *Fusarium graminearum* . Mol Plant-Microbe Interact 20: 627–636. 17555271 10.1094/MPMI-20-6-0627

[56] Gajbhiye A, Rai AR, Meshram SU, Dongre AB (2010) Isolation, evaluation and characterization of *Bacillus subtilis* from cotton rhizospheric soil with biocontrol activity against *Fusarium oxysporum* . World J Microb Biot 26: 1187–1194. 10.1007/s11274-009-0287-9 24026922

[57] Kang Z, Buchenauer H (2000) Cytology and ultrastructure of the infection of wheat spikes by *Fusarium culmorum* . Mycol Res 104: 1083–1093.

[58] Xue A, Voldeng H, Savard M, Fedak G (2009) Biological management of Fusarium head blight and mycotoxin contamination in wheat. World Mycotoxin J 2: 193–201.

[59] Groth JV, Ozmon EA, Busch RH (1999) Repeatability and Relationship of Incidence and Severity Measures of Scab of Wheat Caused by *Fusarium graminearum* in Inoculated Nurseries. Plant Dis 83: 1033–1038. 10.1094/PDIS.1999.83.11.1033 30841272

[60] Maragos CM, Plattner RD (2002) Rapid fluorescence polarization immunoassay for the mycotoxin deoxynivalenol in wheat. J Agric Food Chem 50: 1827–1832. 11902919 10.1021/jf011487d

[61] Liu Y, Walker F, Hoeglinger B, Buchenauer H (2005) Solvolysis procedures for the determination of bound residues of the mycotoxin deoxynivalenol in *Fusarium* species infected grain of two winter wheat cultivars preinfected with barley yellow dwarf virus. J Agric Food Chem 53: 6864–6869. 16104812 10.1021/jf050831u

[62] Bie X, Lu Z, Lu F (2009) Identification of fengycin homologues from *Bacillus subtilis* with ESI-MS/CID. J Microbiol Methods 79: 272–278. 19781583 10.1016/j.mimet.2009.09.013

[63] Snedecor GW, Cochran WG (1989) Statistical Methods. Ames: Iowa State University Press. 503 p.

[64] Stein T (2008) Whole-cell matrix-assisted laser desorption/ionization mass spectrometry for rapid identification of bacteriocin/lantibiotic-producing bacteria. Rapid Commun Mass Spectrom 22: 1146–1152. 18335461 10.1002/rcm.3481

