# Supplementary material for: Antagonistic Action of Bacillus subtilis Strain SG6 on Fusarium graminearum
Source: PLoS One. 2014 Mar 20;9(3):e92486. doi: 10.1371/journal.pone.0092486 (PMC3961383; doi:10.1371/journal.pone.0092486)
Supplement: Table S1 — Information for collected samples. (DOCX) [file pone.0092486.s001.docx]

## Table S1

| Name | Description | Sources | Person contacted for further permission |
| --- | --- | --- | --- |
| W1 | Wheat kernel | private land in Liudi Village, Xiajin Town, Dezhou City, Shandong Province | Liu Dehui |
| W2 | Wheat kernel | private land in Zhangjia Village, Ningjin Town, Rongcheng City, Shandong Province | Zhang Huanhuan |
| W3 | Wheat kernel | private land in Xiashuihe Village, Laichengqukou Town, Laiwu City, Shandong Province, | Zheng Ran |
| W4 | Wheat kernel | private land in Lianjiazhuang Village, Songlindian Town, Zhuozhou City, Hebei Province | Liu Yachang |
| W5 | Wheat kernel | private land in Qinggang Village, Xiahuliang Town, Zhouzhou City, Hebei Province | Cai Shimin |
| W6 | Wheat kernel | private land in Aixin Village, Ningjin Town, Xingtai City, Hebei Province | Zhang Wei |
| W7 | Wheat kernel | private land in Xingsheng Village, Lichuan Town, Enshi City, Hubei Province | Zhou Dazhai |
| W8 | Wheat kernel | private land in Nangang Village, Yuanshi Town, Songzi City, Hubei Province | Zhu Junchang |
| W9 | Wheat kernel | private land in Shiping Village, Datong Town, Qichun City, Hubei Province | He Zhiming |
| W10 | Wheat kernel | private land in Banbidian Village, Daxing District, Beijing | Zhao Huixin |
| L11 | Anthers of luffa | yard of Chinese Academy of Agricultural Sciences, 2 West Yuanmingyuan Road, Beijing. | Yong Zhenguo |
| B12 | Anthers of bean |  |  |
| C13 | Anthers of Chinese Rose |  |  |
| P14 | Anthers of pumpkin |  |  |

Information for collected samples
