# Supplementary material for: Antagonistic Action of Bacillus subtilis Strain SG6 on Fusarium graminearum
Source: PLoS One. 2014 Mar 20;9(3):e92486. doi: 10.1371/journal.pone.0092486 (PMC3961383; doi:10.1371/journal.pone.0092486)
Supplement: Table S2 — Primers used for AMP amplification in this study. (DOCX) [file pone.0092486.s002.docx]

## Table S2

| Primer | Sequence | Product size (bp) |
| --- | --- | --- |
| srfAF | TCGGGACAGGAAGACATCAT | 201 |
| srfAR | CCACTCAAACGGATAATCCTGA |  |
| bmyBF | GAATCCCGTTGTTCTCCAAA | 370 |
| bmyBR | GCGGGTATTGAATGCTTGTT |  |
| bacAF | CAGCTCATGGGAATGCTTTT | 498 |
| bacAR | CTCGGTCCTGAAGGGACAAG |  |
| fenDF | GGCCCGTTCTCTAAATCCAT | 269 |
| fenDR | GTCATGCTGACGAGAGCAAA |  |
| ituCF | GGCTGCTGCAGATGCTTTAT | 423 |
| ituCR | TCGCAGATAATCGCAGTGAG |  |

Primers used for AMP amplification in this study.
